# Frequency and types of motor fluctuations in patients presenting with advanced Parkinson’s disease

**DOI:** 10.12669/pjms.42.7.15138

**Published:** 2026-07

**Authors:** Samiksha, Qamar-Un-Nisa, Muhammad Irfan, Wajid Jawaid, Sumera Rafat Umer, Sidra Jazil Faruqi

**Affiliations:** 1 Samiksha, MBBS, Department of Neurology, Dow University of Health Sciences, Karachi, Pakistan; 2 Qamar-Un-Nisa, FCPS, Department of Neurology, Dow University of Health Sciences, Karachi, Pakistan; 3 Muhammad Irfan, FCPS, Department of Neurology, Dow University of Health Sciences, Karachi, Pakistan; 4 Wajid Jawaid, FCPS, Department of Neurology, Dow University of Health Sciences, Karachi, Pakistan; 5 Sumera Rafat Umer, FCPS, Department of Neurology, Dow University of Health Sciences, Karachi, Pakistan; 6 Sidra Jazil Faruqi, FCPS, Department of Neurology, Dow University of Health Sciences, Karachi, Pakistan

**Keywords:** Dyskinesia, Levodopa complications, Motor Fluctuations, Parkinson’s Disease, Wearing-off

## Abstract

**Objective:**

To determine the frequency and types of motor fluctuations in patients presenting with advanced Parkinson’s disease at a tertiary care hospital in Pakistan.

**Methodology:**

This descriptive cross-sectional study was conducted at the Department of Neurology, Dr. Ruth K.M Pfau Civil Hospital, Karachi, from 1st June 2025 to 30th November 2025. A total of one hundred patients aged 25-75 years with advanced Parkinson’s disease were enrolled through consecutive non-probability sampling. Demographic, clinical, and disease-related variables were documented, motor fluctuations were systematically assessed using clinical impression of severity index for Parkinson disease (CISI-PD), and data were analyzed in SPSS v26 with Chi-square tests (p ≤ 0.05).

**Results:**

In this cohort of one hundred advanced Parkinson’s disease patients, the mean age was 49.9 years (±15.6), with a mean BMI of 27.5 (±8.7) and average disease duration of 7.2 years (±11.9). The sample included 51% men and 49% women. Motor fluctuations were reported in 42%, predominantly wearing off (28.6%) and dyskinesia (26.2%). No demographic, clinical, or treatment factor showed significant association (p>0.05), though smokers exhibited a higher frequency (51.2% vs.35.1%, p = 0.080).

**Conclusion:**

Wearing-off episodes and dyskinesia were the most common fluctuations in this study.

## INTRODUCTION

Parkinson’s disease (PD), first described by James Parkinson in 1817, is a progressive neurodegenerative disorder that significantly impairs motor and non-motor functioning, leading to reduced quality of life for affected individuals. It is recognized as the second most prevalent age-related neurodegenerative condition after Alzheimer’s disease, with a global prevalence of approximately 1% among those over 60 years of age and up to 5% in individuals over 85.^1^–^3^ The disease is characterized by motor symptoms such as: Wearing off, dyskinesia, on-off phenomenon and non-motor symptoms such as: constipation, sleep behavior disorder (RBD), olfactory dysfunction (hyposmia).^4^ The burden of PD continues to rise worldwide, reflecting aging populations and greater awareness of diagnostic features, though marked variation exists across geographic, ethnic and socioeconomic group.^5^,^6^

Although the aetiology of PD remains multifactorial, with genetic, environmental, and occupational exposures contributing to risk, men are consistently reported to have higher prevalence than women.^7^,^8^ The cornerstone of symptomatic management remains dopaminergic replacement therapy, particularly levodopa, which provides substantial improvement in motor function. However, long-term treatment is commonly complicated by motor fluctuations, such as wearing-off, dyskinesias, on–off episodes, and freezing phenomenon.^9^

Evidence from longitudinal studies, including the CamPaIGN and DATATOP cohorts, has demonstrated that motor fluctuations are almost inevitable with prolonged levodopa exposure.^9^,^10^ Despite extensive data from high-income countries, there remains a paucity of literature from low and middle-income countries (LMICs), where treatment access, prescribing practices, and health-seeking behaviors may differ substantially. This gap is particularly evident in South Asia, including Pakistan, where little is known about the burden and clinical correlates of motor complications in advanced PD.

Understanding the prevalence and patterns of motor fluctuations in this context is critical for two reasons. First, it provides insights into the natural course of disease progression in populations with distinct demographic and environmental exposures. Second, it informs clinicians and policy makers about the need for early recognition and timely management strategies, especially in healthcare systems with limited resources and fewer advanced treatment options such as device-assisted therapies.

In light of these considerations, this study was designed to determine the frequency and patterns of motor fluctuations in patients with advanced Parkinson’s disease presenting to a tertiary care hospital in Pakistan. By documenting the prevalence of motor complications and exploring their associations with demographic and clinical characteristics, this research aims to fill an important regional knowledge gap and provide evidence that may guide both clinical practice and health policy in the management of advanced PD.

## METHODOLOGY

This was a cross-sectional study conducted at Department of Neurology at Dr. Ruth K.M Pfau Civil Hospital, Karachi, spanning the period from 1st June 2025 to 30th November 2025. A total of one hundred participants were enrolled from the outpatient neurology services utilizing a consecutive non-probability sampling methodology, with the sample size determined employing the WHO calculator predicated on an anticipated prevalence of motor fluctuations at 40%, a confidence level of 95%, and a margin of error of 10%.^10^ Patients of either gender, aged 25–75 years, with a confirmed diagnosis of advanced Parkinson’s disease were included.

### Ethical Approval:

This study was approved by the Institutional Review Board (IRB) committee of Dow University of Health Sciences on May 29, 2025 (IRB-3975/DUHS/Approval/2025/203).

Advanced Parkinson’s disease was defined as the presence of significant disability despite levodopa therapy with at least one of the following: postural instability, freezing episodes, severe dysphagia, or complications such as psychosis, or dementia. Wearing-off was defined as predictable recurrence of symptoms before the next scheduled dose. On-off fluctuations were defined as abrupt unpredictable transitions between mobile and immobile states. Freezing phenomenon was defined as transient inability to initiate or continue movement despite intention to walk. Delayed-on referred to delayed onset of medication benefit after dosing. End-of-dose worsening referred to symptom deterioration near completion of medication effect. Dyskinesias were categorized as off-period, diphasic, or peak-dose involuntary movements.^11^ Patients with supranuclear palsy, vascular parkinsonism, drug-induced parkinsonism, multiple system atrophy, malignancy, or pregnancy were excluded. The exposure variable was advanced Parkinson’s disease, while the outcome variable was the presence of motor fluctuations, defined as oscillations in motor response, including wearing-off, on–off fluctuations, freezing phenomenon, delayed on, end-of-dose worsening, and dyskinesias (off-period, diphasic, or peak- dose).

After obtaining informed consent, demographic and clinical data were recorded using a structured proforma, including name, age, gender, body mass index, residence, occupation, socioeconomic status. Motor fluctuations were identified through detailed clinical assessment by the investigator during patient interviews and neurological examination, supplemented by review of treatment history and symptom patterns. Socioeconomic status was categorized using household-income-based fixed cutoffs (with <35,000 PKR categorized as low socioeconomic status, 35,000 -80,000 PKR as middle, and > 80,000 PKR as high socioeconomic status), smoking history (>5 pack-years), alcohol intake (>20 ml/day), diabetes (random blood sugar >200 mg/dl), hypertension (blood pressure ≥140/90 mm Hg), and family history of Parkinson’s disease. Disease-related variables such as duration of Parkinson’s disease, treatment regimen and compliance were derived through careful history. Treatment response was assessed using subjective patient-reported outcomes, including functional improvement, daily activity performance, and symptoms relief. Each patient was systematically assessed for the presence, types, and onset of motor fluctuations, and disease severity was evaluated using the Clinical Impression of Severity Index for Parkinson’s Disease (CISI-PD).^12^

All assessments were performed by the investigators to ensure uniformity, and patients were managed as per institutional protocols. Data were analyzed using SPSS version 26; descriptive statistics summarized baseline variables, and associations between motor fluctuations and patient characteristics were assessed using the Chi-square test, with p ≤ 0.05 considered statistically significant.

## RESULTS

One hundred patients with advanced Parkinson’s disease were included in the analysis. The study population showed a nearly equal gender distribution, with 51% males and 49% females. Most patients were receiving polytherapy (75%), while levodopa monotherapy was used by 25% of participants. Diabetes mellitus was the most common comorbid condition, present in 44% of the study population. Detailed demographic characteristics, treatment patterns, and clinical characteristics are presented in Tables-I, II, and III, respectively.

**Table-I:** Demographic Profile of the Study Population.

Mean ± Standard Deviation		95% Confidence Interval
Age in years = 49.86 ± 15.58		46.77―52.95
BMI = 27.45 ± 8.70		25.72―29.18
Duration of Parkinson’s Disease in years = 7.15 ± 11.92		6.46―29.18
***Frequency (%)***		
Gender	Male	51 (51.0)
	Female	49 (49.0)
Resident	Urban	57 (57.0)
	Rural	43 (43.0)
Occupation	Employed	62 (62.0)
	Unemployed	38 (38.0)
Socioeconomic Status	Low	31 (31.0)
	High	22 (22.0)
	Middle	47 (47.0)

**Table-II:** Oral drugs taken by study population.

Treatment	*Monotherapy*	*Polytherapy*
	Levodopa (25%)	Levodopa + Amantadine (35%)
		Levodopa + Ropinirole (20%)
		Levodopa + Procyclidine (6%)
		Levodopa + Amantadine + Ropinirole (14%)

**Table-III:** Clinical Characteristics of Study Participants (n=100).

Smoking	Yes	43 (43.0)
	No	57 (57.0)
Alcoholism	Yes	0 (0.0)
	No	100 (100.0)
Diabetes	Yes	44 (44.0)
	No	56 (56.0)
Hypertension	Yes	27 (27.0)
	No	73(73.0)

When evaluating motor complications, wearing-off episodes (28.6%) and dyskinesias (26.2%) were the leading contributors. Other fluctuation patterns included on–off phenomena (19.0%), delayed-on (11.9%), freezing phenomenon (9.5%), and peak-dose effects (4.8%).

When comparing participants with and without motor fluctuations, no statistically significant associations were observed across demographic or clinical variables. The mean age of patients who experienced motor fluctuations was 47.6 ± 16.6 years, compared to 51.5 ± 14.7 years in those without fluctuations (p = 0.398). Similarly, the mean duration of Parkinson’s disease was slightly longer in patients with fluctuations (7.4±3.5years) than in those without (7.0±3.1years), though this difference was not significant (p=0.572). Gender distribution was comparable between groups, with 41.2% of males and 42.9% of females experiencing motor fluctuations (p=0.865). A similar trend was observed with place of residence: 42.1% of urban residents and 41.9% of rural residents reported fluctuations (p=0.980). Occupational status did not show a significant association either, as 43.5% of employed individuals and 39.5% of unemployed individuals experienced motor fluctuations (p=0.689).

Socioeconomic status was also not significantly related to fluctuations Motor complications were observed in 41.9% of patients in the low-income group, 45.5% in the high-income group, and 40.4% in the middle-income group (p = 0.925). Among lifestyle factors, a higher proportion of smokers (51.2%) developed motor fluctuations compared to non-smokers (35.1%), although this association did not reach statistical significance (p=0.080).

Comorbidities showed no significant correlation with motor fluctuations. 47.7% of patients with diabetes experienced fluctuations versus 37.5% without diabetes (p=0.205). Similarly, 51.9% of hypertensive patients reported fluctuations compared to 38.4% without hypertension (p=0.162). In terms of treatment regimens, 30% of patients received levodopa alone, while 70% were receiving polytherapy, most frequently levodopa + ropinirole (40%), followed by levodopa + amantadine (6%), levodopa + procyclidine (6%), and levodopa + amantadine + ropinirole (18%) (p=0.651). Overall, none of the examined demographic, clinical, or treatment-related characteristics demonstrated a statistically significant relationship with the occurrence of motor fluctuation.

## DISCUSSION

The present investigation evaluated the occurrence and distribution of motor fluctuations among individuals with advanced Parkinson’s disease managed at a tertiary hospital in Pakistan. We observed that 42% of patients experienced motor fluctuations. Among the various motor fluctuation patterns identified, wearing-off episodes (28.6%) and dyskinesias (26.2%) were the most frequently observed.

Other forms such as on–off fluctuations, delayed-on, freezing phenomenon, and peak-dose effects were less frequent. These complications represent a defining feature of advanced disease, affecting nearly half of patients within five years of treatment initiation and the majority after a decade.^13^,^14^

The studies conducted in China showed a higher prevalence of wearing-off (46.5%) but a lower rate of dyskinesia (10.3%).^15^,^16^ Whereas studies conducted in Europe and South Korea reported different results, with wearing-off around 31.2% and dyskinesia about 27.6%.^17^-^19^

Wearing-off was the most frequently reported complication, consistent with the Wearing-Off Questionnaire validation study, which highlighted it as the earliest and most common clinical manifestation of treatment-related decline.^20^ Dyskinesia, found in one-quarter of patients, is similarly well documented in longitudinal cohorts such as CamPaIGN, where it has been shown to steadily increase with disease progression.^10^

The overall prevalence of motor fluctuations in our cohort aligns with rates reported internationally. Long-term studies have consistently demonstrated that complications of levodopa therapy become almost inevitable with time, affecting approximately half of patients within five years and up to 90% after a decade of treatment exposure.^21^

Risk factors such as younger age at onset, longer disease duration, higher cumulative doses, and non-tremor dominant phenotypes have been implicated in the earlier appearance of these complications.^22^,^23^ Cross-sectional studies conducted in China and Europe have reported prevalence estimates ranging between 35% and 50%.^14^ which are comparable to the 42% documented in our study.

Notably, no significant association was observed between motor fluctuations and demographic or treatment-related factors in our analysis. While some studies have identified younger age at onset, longer duration of illness, and higher cumulative levodopa dose as predictors of early motor complications.^22^ These associations were not evident in our sample.

This may be explained by the modest sample size, which limited statistical power, or by differences in prescribing practices, cultural health-seeking behaviors and the stage at which patients present to tertiary centers in Pakistan. Previous research has considered environmental, and lifestyle influences on Parkinson’s disease progression, but findings remain inconsistent.^8^ Larger multicenter studies could provide greater clarity on whether smoking acts as a risk factor in this context.

### Strengths

It addresses an important gap in literature providing systematic, region-specific evidence on motor fluctuations in advanced Parkinson’s disease, an area where data from South Asia are extremely limited. The use of standardized definitions for advanced disease and structured clinical assessments ensured diagnostic consistency. Furthermore, the collection of comprehensive demographics, clinical and therapeutic data allowed for a wide-ranging evaluation of potential correlates.

### Limitations

The cross-sectional nature of the study restricts causal interpretation and prevents exploration of temporal predictors of motor complications. The relatively small sample size may have reduced the likelihood of detecting significant associations in subgroup analyses, particularly with lifestyle factors and comorbidities. Information regarding cumulative drug dose, treatment duration, and dosing frequency was not systematically analyzed and may have influenced the occurrence of motor fluctuations. The study relied primarily on descriptive and univariate analyses. Multivariable analysis could not be performed because of sample size considerations. Future studies with larger cohorts should employ multivariate analyses to identify independent predictors of motor fluctuations. Additionally, recruitment from a single tertiary hospital may limit external validity, as patients in such facilities often represent more severe or complex cases compared to those managed in community settings.

Finally, although clinical assessment is practical, it may not fully capture subtle or intermittent motor fluctuations that could be identified with validated diaries or digital monitoring tools.^20^ Limited access to advanced PD therapies in Pakistan highlights the need for early pharmacological optimization and structured follow-up.^24^

## CONCLUSION

Motor fluctuations represent a prominent challenge in advanced Parkinson’s disease, with patterns such as wearing-off and dyskinesia being most notable. The absence of clear demographic or treatment-related associations in this cohort suggests that these complications may be intrinsic to disease progression. Recognizing and addressing them early is essential for optimizing care and improving quality of life, particularly in resource-constrained healthcare environments.

### Recommendations

Future multicenter studies involving larger patient populations should evaluate predictors of motor fluctuations like cumulative levodopa exposure, disease phenotype, medication dosing patterns, and quality-of-life outcomes. Longitudinal studies would also help clarify temporal relationships and progression of motor complications.

### Author’s contribution:

**S:** Concept design, data, collection, manuscript writing.

**QUN** and **MI**: Design, data, analysis, manuscript revision.

**WJ:** Design, data interpretation, manuscript writing.

**SRU** and **SJF:** Data analysis, manuscript, revision.

**S**: Responsible and accountable, accuracy and integrity of the study.
